# A Microsurgical Arteriovenous Malformation Model on Saphenous Vessels in the Rat

**DOI:** 10.3390/biomedicines11112970

**Published:** 2023-11-04

**Authors:** Mohammad Walid Al-Smadi, Laszlo Adam Fazekas, Siran Aslan, Brigitta Bernat, Anas Beqain, Mustafa Qais Muhsin Al-Khafaji, Daniel Priksz, Brigitta Orlik, Norbert Nemeth

**Affiliations:** 1 Department of Operative Techniques and Surgical Research, Faculty of Medicine, University of Debrecen, Moricz Zsigmond ut 22, 4032 Debrecen, Hungary; m.smadi@med.unideb.hu (M.W.A.-S.); fazekas.laszlo@med.unideb.hu (L.A.F.); aslan@mailbox.unideb.hu (S.A.); beqain@mailbox.unideb.hu (A.B.); mustafaqais@mailbox.unideb.hu (M.Q.M.A.-K.); 2 Kalman Laki Doctoral School, University of Debrecen, 4032 Debrecen, Hungary; 3 Department of Pharmacology and Pharmacotherapy, Faculty of Medicine, University of Debrecen, Nagyerdei krt. 98, 4032 Debrecen, Hungary; bernat.brigitta@med.unideb.hu (B.B.); priksz.daniel@pharm.unideb.hu (D.P.); 4 Department of Pathology, Faculty of Medicine, University of Debrecen, Nagyerdei krt. 98, 4032 Debrecen, Hungary; orlik.brigitta@med.unideb.hu

**Keywords:** arteriovenous malformation, arteriovenous shunt, microsurgical model, vascular remodelling, hemodynamics

## Abstract

Arteriovenous malformation (AVM) is an anomaly of blood vessel formation. Numerous models have been established to understand the nature of AVM. These models have limitations in terms of the diameter of the vessels used and the impact on the circulatory system. Our goal was to establish an AVM model that does not cause prompt and significant hemodynamic and cardiac alterations but is feasible for follow-up of the AVM’s progression. Sixteen female rats were randomly divided into sham-operated and AVM groups. In the AVM group, the saphenous vein and artery were interconnected using microsurgical techniques. The animals were followed up for 12 weeks. Anastomosis patency and the structural and hemodynamic changes of the heart were monitored. The hearts and vessels were histologically analyzed. During the follow-up period, shunts remained unobstructed. Systolic, diastolic, mean arterial pressure, and heart rate values slightly and non-significantly decreased in the AVM group. Echocardiogram results indicated minor systolic function impact, with slight and insignificant changes in aortic pressure and blood velocity, and minimal left ventricular wall enlargement. The small-caliber saphenous AVM model does not cause acute hemodynamic changes. Moderate but progressive alterations and venous dilatation confirmed AVM-like features. The model seems to be suitable for studying further the progression, enlargement, or destabilization of AVM.

## 1. Introduction

Arteriovenous malformations (AVMs) are vascular anomalies composed of a feeding artery and a draining vein without an intervening network of capillaries [1,2,3]. This allows a high blood flow to connect the two, forming complex, tangled vessels called a *nidus* [4,5,6]. Some studies suggest that AVMs arise during embryogenesis, particularly in the third week of development, where the primordial vasculature fails to mature into a proper capillary network [7,8,9]; however, AVMs can also arise postnatally and may grow after resection or radiotherapy [10,11].

AVMs may result in various symptoms depending on their locations. Due to the high arterial pressure flow through the delicate vessels in the nidus and the draining veins, the vessels are susceptible to ruptures, leading to disability or even death from intracranial hemorrhage [11,12,13]. Moreover, AVM can present with other neurological diseases, ranging from chronic headaches and migraines to epileptic seizures and ischemic strokes [14,15,16,17]. Furthermore, AVM may occur extracranially in the spine, head, neck, limbs, and organs [18,19,20,21,22].

The diagnosis of cerebral AVMs is usually incidental or after a workup of acute cerebral hemorrhage or during an investigation of unexplained seizures or chronic headaches [16]. AVMs can be in different sizes and locations and have various feeding and draining vessels. Therefore, multiple scales have arisen to describe and classify them. The most common classification for AVMs is the Spetzler–Martin score, which grades AVMs based on size, location, and venous drainage pattern. A higher score reflects a greater risk of morbidity and mortality in microsurgical removal [23]. In 2010, a supplementary scale was added to the Spetzler–Martin scale, making a 10-point scale by combining Spetzler––Martin with age, hemorrhagic presentation, and lesion nidus diffuses [24,25,26]. The Toronto scale is another nine-point scale that predicts neurological outcomes after AVM resection [27]. 

Depending on the AVM grade and the previously mentioned scales, multidisciplinary strategies are followed, including radiosurgery for smaller lesions to microsurgical resection and endovascular embolization [13,28,29,30]. Surgical excision remains the gold standard for most lesions’ drastic and definitive elimination [28]. However, about 25% of patients experience a recurrence of AVMs during the first year following intervention due to reperfusion brought on by recanalization, previously undetected feeding, or neighboring arteries [31,32].

Despite substantial advancements in basic research, clinical care, and applying technology in medicine, AVM management remains one of the mysteries yet to be solved. This stems from our poor understanding of the AVM’s behavior with or without interventions. Earlier, it was supposed that small-size AVMs have a higher risk for bleeding because of the stronger arterial feeding pressure [24], while other recent studies did not support this theory [33]. It is still not completely known how the hemodynamic forces formulate the shape of the AVMs and the possible progression to rupture with and without an aneurysm [34,35,36]. Various models have been created to understand AVMs’ pathogenesis, behavior, and response to treatment [37,38]. Some used gene manipulation [39,40,41], and others used surgical vascular anastomosis models [42,43,44,45,46,47,48]. These models have faced various challenges in resembling AVMs’ anatomy, physiology, biology, and clinical features for human AVMs, which is why there is a need for an ideal AVM model that gives a better understanding of AVMs as an excellent analogy. 

We hypothesized that a peripheral, small-caliber, microsurgically created arteriovenous shunt in a fixed geometry that does not cause prompt, acute changes in hemodynamics and cardiac function compared to other models would be asymptomatic for a longer time but the progression of enlargement can be monitored over several months of the follow-up period. In this study, we aimed to create an AVM model by utilizing saphenous vessels in rats, examining the morphology and systemic effects on the circulatory system, and investigating the local vascular changes (remodeling) on the vessels.

## 2. Materials and Methods

### 2.1. Experimental Animals

The University of Debrecen’s Committee of Animal Welfare (UDCAW) and the National Food Chain Safety Office registered and approved this study (registration Nr. 25/2016/UDCAW, 25/2022/UDCAW) in accordance with the national (Act XXVIII of 1998 on the protection and sparing of animals) and EU (Directive 2010/63/EU) regulations. Sixteen female outbred CD^®^ (Sprague Dawley) rats (Charles River Laboratories International, Inc.; Wilmington, MA, USA) 12-week old, bodyweight: 293.21 ± 10.33 g) were involved in the study. They were randomly divided into sham-operated control group (Sham, *n* = 8), and arteriovenous malformation group (AVM, *n* = 8). The rats were housed in a conventional microbiological status animal facility in standard cages (1354G Eurostandard Type IV, 595 × 380 × 200 mm, floor area 1820 cm^2^, Tecniplast S.p.A., Buguggiate, Italy), in alternating day and night light conditions in a 12 h cycle, and were given unrestricted access to commercially available rodent chow (SAFE^®^ D132 autoclavable complete universal vegetal diet for rats, mice, and hamsters) and tap water.

### 2.2. Operative Techniques

Rats were anesthetized using a mixture of ketamine (100 mg/kg), atropine (0.05 mg/kg), and xylazine (10 mg/kg) intraperitoneally [49]. In addition, to prevent thrombotic events, the rats received an intravenous injection of heparin (80 IU/kg). In the case of perioperative medication requirements, a secure 26-gauge cannula was installed in the lateral vein of the tail. 

**Figure 1:**
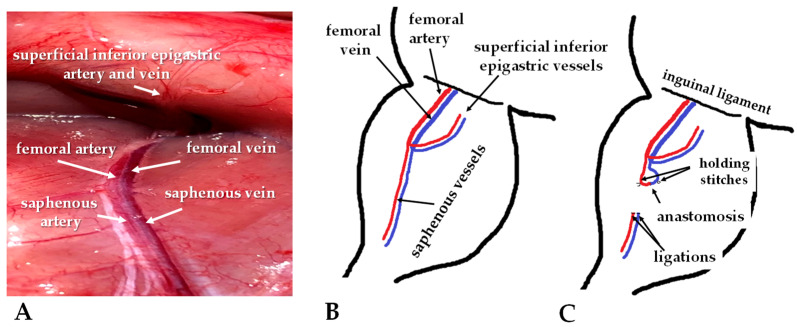
Intraoperative photos of the intact vessels (**A**), schematic drawing of the affected vessels (**B**), and the localization of the anastomoses (**C**).

**Figure 2:**
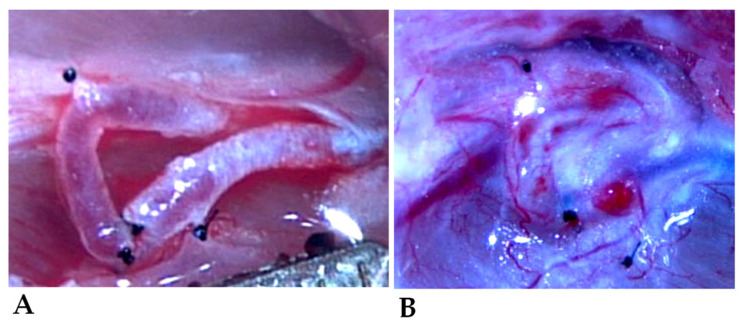
Intraoperative view (captured from video recording) of the freshly performed shunt (**A**), and the picture on the 12th postoperative week (**B**).

For the operation, sterile instruments were used. The lower abdomen, inner thighs, and upper legs were shaved and disinfected with Betadine; the operation site was isolated with sterile gauze. A 3-centimeter oblique incision was made on the middle of the thigh region. In the AVM group, the intervention was performed on the right saphenous veins and arteries with diameters ranging between 0.3 and 0.4 mm (Figure 1 and Figure 2).

Atraumatic microsurgical dissection was carried out to expose the vessels. Distal ligation and proximal clipping of both the artery and vein were conducted. The vein was cut more distally than the artery to provide space and flexibility for subsequent surgical steps. The vessels were washed with heparin and carefully mobilized to the appropriate position.

Three 3/8 serosa (taper) needles with 10/0 non-absorbable monofilament polyamide-6 suture material (Daclon, SMI, Saint Vith, Belgium) were utilized simultaneously for successful end-to-end anastomosis. This approach was found to be less traumatic and time-efficient, given the small size and tendency of the vessels to collapse. The first needle was used to place the initial suture in the 12 o’clock position in both vessels without knotting. Then, the needle was inserted into the opposite end of the vessel, the 6 o’clock position, and the same step was repeated, resulting in a wrapped configuration. Next, the third needle was inserted mid-portion between the first two entry points. After securing the three threads, they were tied and knotted to close the front wall of the anastomosis. Next, the vessels were flipped, and a final fourth knot was placed in the middle of the back wall, completing the anastomosis (Figure 2).

Upon completion of the anastomosis, we tested for leakage. The proximal microvascular clip was removed from the venous side, and any flaws in the anastomosis were observed. If no issues were detected, the arterial microvascular clip was removed, and subcutaneous fat was temporarily placed on the anastomosis to improve coagulation if needed. The patency of the anastomosis was assessed using the double occlusion test or the ‘milking test’ [50]. Finally, a continuous suture using a 4/0 absorbable polyglecaprone suture material (Simfra Sutures, Kollsut, Miami, FL, USA) with a 3/8 cutting needle was employed for skin closure.

Following anesthesia, a similar skin incision was made for the sham-operated group, followed by dissection. The rats remained under sedation for about 60 min, equivalent to the time required to complete the anastomosis in the AVM group. The skin closure for the sham-operated group was performed similarly to the AVM group.

On the first 3 postoperative days Tramadol was given at 15 mg/bwkg, i.p., for analgesia [51]. During the follow-up period of 12 weeks, regular observation and wound care were taken. 

### 2.3. Ultrasound Examination

On the 3rd, 7th, and 12th postoperative week, echocardiography was carried out using Vevo 3100 Ultrasound Imaging System (Fujifilm VisualSonics Inc., Toronto, ON, Canada) equipped with a high-frequency transducer (MX250, 14–28 MHz). In the morning hours, rats were anesthetized intraperitoneally with ketamine-xylazine solution (50/5 mg/kg), then shaved and placed onto a real-time electrocardiogram (ECG) and heart rate (HR) monitoring platform (VisualSonics SR200). 

Cardiac function was evaluated in accordance with the recommendations of the American Society of Echocardiography [52]. The database was built from B-, 2D-, M-, and Doppler modes, from parasternal long- and short-axes, as well as from suprasternal (aortic arch) and apical four-chamber views (PSLAX, PSAX, SST, and A4C, respectively). Following acquisitions, data were analyzed offline by a blinded reader using the VevoLAB software (version 5.1, Fujifilm VisualSonics Inc., Toronto, ON, Canada). Heart rate was automatically calculated from R–R intervals of a 5-second period of the recorded ECG. Wall thickness and chamber diameters were measured in M-mode, from PSLAX and PSAX views, at the mid-level of the papillary muscles. Left atrial (LA) maximal diameter (mm) and aortic root (Ao) diameter (mm) were also determined from M-mode images. Left ventricle (LV) internal diameter in end-diastole (LVIDd, mm) and end-systole (LVIDs, mm), as well as anterior and posterior LV wall thickness (mm, LVAWd, LVAWs, and LVPWd, LVPWs, respectively) were measured using M-mode tracing in PSLAX view. Left ventricular volume in end-diastole and end-systole (LVVOLd and LVVOLs, respectively; µL) were calculated using the software. The left ventricular ejection fraction (LVEF, %) was calculated as 100*(LVVOLd-LVVOLs)/LVVOLd. Cardiac output (CO, mL/min) was automatically calculated as SV*HR, and stroke volume (SV, L) as LVIDd-LVIDs. 

Diastolic function was evaluated using pulsed-wave Doppler (PW) and tissue Doppler imaging (TDI) from apical four-chamber views. PW Doppler echocardiography was used to measure the ratio of the transmitral early (E) and atrial (A) peak flow velocities (E/A ratio) and the E wave deceleration time (DecT, ms). Mitral valve closure to opening time (MCOT, ms), isovolumic contraction time (IVCT, ms), left ventricular ejection time (LVET, ms), and isovolumic relaxation time (IVRT, ms) were determined using PW Doppler imaging in the left ventricular cavity. Myocardial performance index (MPI) was calculated as (MCOT-LVET)/LVET. Aortic flow (Ao) parameters were also assessed in the PW Doppler mode from the modified suprasternal view. Aortic velocity time integral (Ao VTI, mm), aortic flow velocities (Ao mean Vel, Ao peak Vel, mm/s), and pressure gradients (Ao mean Grad, Ao peak Grad, mmHg) were measured using the software after manually tracing the borders of the Doppler jet. The pulmonary vein was visualized using a modified PSLAX view [53]. Pulmonary vein systolic (PV S, mm/s) and diastolic (PV D, mm/s) deflections and PV atrial reversal peak velocity (PV Ar, mm/s) and duration (PV ARdur, ms) were measured. Tissue Doppler imaging (TDI) was performed at the septal annulus to evaluate peak tissue velocities at systole (s’, mm/s), and in early (e’ mm/s) and late (a’, mm/s) filling. Wall motion velocity was defined as the e’/a’ ratio. The ratio of E/e’ was also determined. Three cardiac cycles were averaged for each parameter, data are presented as the mean ± S.D.

### 2.4. Hemodynamic Measurements

Twelve weeks after the surgery, under general anesthesia, the left femoral artery was cannulated for direct hemodynamic measurements: heart rate [bpm], systolic and diastolic blood pressure [mmHg], and calculating mean arterial pressure [mmHg] using the Hemosys monitoring system (Experimetria Ltd., Budapest, Hungary). The anastomoses were checked for patency (‘milking test’). After the hemodynamic measurements, the animals were over-anesthetized.

### 2.5. Histological Investigation

Using an operating microscope, the histological samples—including the hearts and vessels—were dissected and fixed in 4% formalin for one week. After fixation, the samples were embedded in paraffin and sectioned (5 µm thickness). Subsequently, hematoxylin and eosin (H&E) and Van Gieson stainings were performed on the sections. The stained slides were then scanned (VENTANA DP200, Hoffmann-La Roche, Roche Holding AG, Basel, Switzerland) to analyze the morphology of the heart, aiming to identify any structural and morphological differences in the cardiomyocytes and blood vessels between the two groups.

To ensure a thorough examination of the histological slides, measurements were taken in three distinct sections of the heart: basal, middle, and apex. The study involved two groups: the control group (consisting of healthy hearts, *n* = 8, and intact saphenous vessels, *n* = 12) and the AVM group (comprising hearts, *n* = 8, intact saphenous vessels, *n* = 8, and anastomosed saphenous vessels, *n* = 8).

For each animal heart, the diameter of the left ventricle’s wall was measured (*n* = 4), and 100 cardiomyocytes were assessed in each section (*n* = 3/animal), resulting in 12 wall diameters and 300 cardiomyocytes per animal heart. These measurements were taken at various locations around the left ventricle, with the cells examined horizontally and their widths measured after zooming in.

In addition to the heart analysis, both the control and AVM groups extracted saphenous vessels from both the right operated leg and the non-operated left leg. In addition, the anastomosis site was examined in the AVM group. Similarly, saphenous vessels were obtained from both legs in the control group. Vessel samples were dissected along with the surrounding tissue to preserve their geometry. In each vessel, ten measurements were taken for each of the three layers: tunica intima, tunica media, and tunica externa. This resulted in a total of 30 measurements per histological slide.

The histological slides of the hearts and blood vessels were evaluated manually using open-source software QuPath 0.4.4 and ImageJ, which enabled precise measurements. The resulting data were recorded in an Excel sheet and numerically analyzed.

### 2.6. Statistical Analysis

Mead’s resource equation method was used to determine the sample size. The statistical analysis was carried out using GraphPad Prism 9 software with a significance level set at *p* ≤ 0.05. Data distribution with normality test was checked for all comparisons. Depending on the normality of data distribution, for inter-group comparisons at a given time point, Student’s *t*-test or Mann–Whitney RS test were used, and for intra-group comparisons, Kruskal–Wallis and Dunn’s method of ANOVA tests were applied.

## 3. Results

### 3.1. Echocardiography

**Figure 3:**
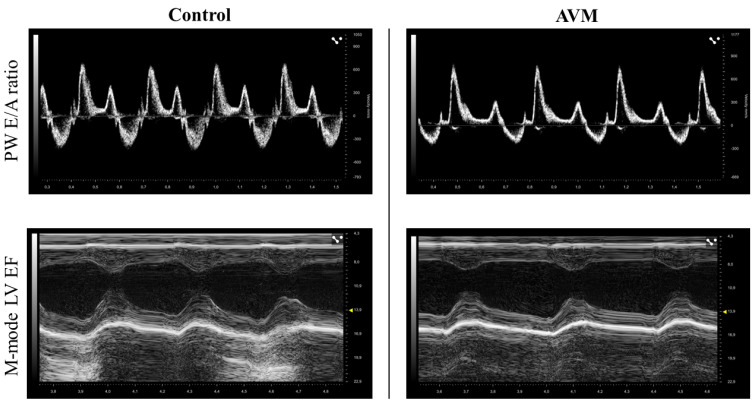
Representative echocardiography recordings of the control and the AVM groups.

Ultrasound assessments of the left ventricle (LV) were conducted preoperatively and during the 3rd, 7th, and 12th postoperative weeks. Figure 3 shows the representative recordings from both groups.

Left ventricle ultrasound analysis disclosed the mass and dimensions of the anterior and posterior walls in systole and diastole. Bodyweight also increased by the 12th p.o. week in both groups as rats were aging (control group: 18.56 ± 6.16%, AVM group: 16.56 ± 3.22%).

**Figure 4:**
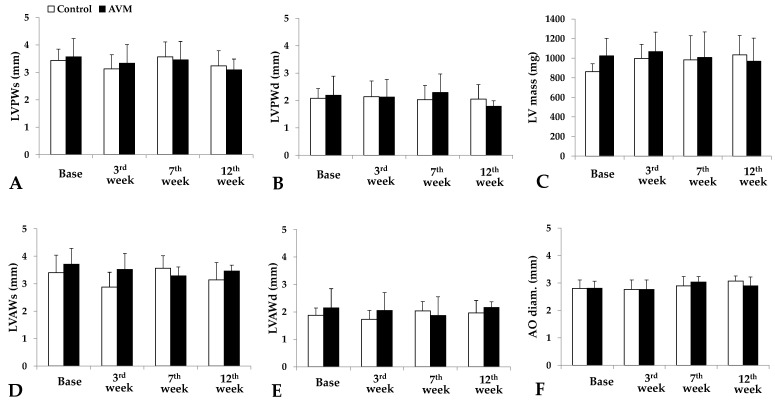
Parameters obtained from the echocardiography recordings preoperatively and on the 3rd, 7th, and 12th postoperative weeks: thickness of the left ventricle posterior wall in systole (LVPWs [mm]) (**A**) and in diastole (LVPWd [mm]) (**B**), the left ventricular mass (LV mass [mg]) (**C**), the thickness of the left ventricle anterior wall in systole (LVAWs [mm]) (**D**) and in diastole (LVAWd [mm]) (**E**), and the aorta diameter (AO diam. [mm]) (**F**). Means ± S.D.

Concerning the posterior wall during systole, the control group demonstrated an initial decrease in thickness from 3.43 ± 0.41 mm to 3.13 ± 0.51 mm, followed by a peak at 3.57 ± 0.54 mm and a subsequent reduction to 3.24 ± 0.55 mm. The AVM group exhibited a similar pattern, with an initial decrease from 3.57 ± 0.54 mm to 3.33 ± 0.68 mm, an increase to 3.46 ± 0.67 mm, and a final decline to 3.09 ± 0.39 mm (Figure 4A). For the posterior wall during diastole, thickness remained relatively stable throughout the study in both the control and AVM groups (Figure 4B).

The systolic thickness of the anterior wall in the control group decreased during the 3rd postoperative week, followed by a return to baseline values later in the study. In the AVM group, the thickness initially diminished from 3.72 ± 0.57 mm preoperatively to 3.29 ± 0.32 mm in the 7th postoperative week, then increased to 3.47 ± 0.21 mm by the 12th week (Figure 4D). Concerning the diastolic thickness of the anterior wall, the control group exhibited a peak in thickness in the 7th postoperative week and a subsequent decline during the 12th week. Meanwhile, the AVM group displayed minimal fluctuations, maintaining a consistency of approximately 2 ± 0.13 mm (Figure 4E).

In the control group, the LV mass demonstrated a mild elevation from 863.15 ± 78.7 mg preoperatively to 997.8 ± 143.4 mg in the 3rd postoperative week, remaining approximately constant for the remainder of the study period. In contrast, the AVM group’s mass persisted at around 1000 ± 41.1 mg during the follow-up period (Figure 4B). Aortic diameter increased in both groups as the animals aged (Figure 4F).

**Figure 5:**
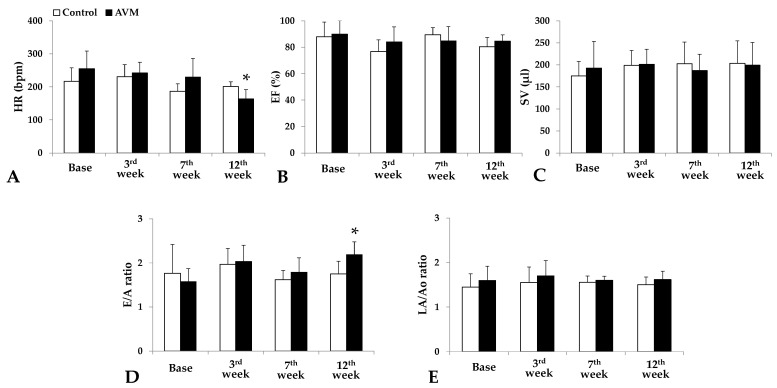
Parameters obtained from the echocardiography recordings preoperatively and on the 3rd, 7th, and 12th postoperative weeks: heart rate (HR [bpm]) (**A**), ejection fraction (EF [%]) (**B**), stroke volume (SV [μL]) (**C**), ratio of the early and atrial peak mitral inflow velocities (E/A ratio) (**D**), and the size of the left atrium normalized to the aortic diameter aorta diameter (LA/Ao ratio) (**E**). Means ± S.D. * *p* < 0.05 vs. Base.

Heart rate slightly increased in the control group from 217 ± 40.5 min^−1^ preoperatively to 231 ± 36.32 min^−1^ during the 3rd week, with a subsequent decrease to 200.8 ± 14.68 min^−1^ by the 12th week. In the AVM group, the value began at 255 ± 53.66 min^−1^ and gradually decreased throughout the study period to 164.2 ± 27.71 min^−1^ (Figure 5A).

Ejection fraction in the control group commenced at 88.03 ± 11.1%, experienced a 10-point drop in the 3rd week, peaked at 89.5 ± 5.29%, and subsequently declined to 80.4 ± 6.93%. In the AVM group, the ejection fraction began at 89.85 ± 10.21% and decreased in the PO weeks to 84.09 ± 11.37%, 84.07 ± 10.82%, and 84.58 ± 4.76% (Figure 5B). 

Regarding stroke volume, the control group exhibited a steady increase from 175.2 ± 32.8 µL to 198.7 ± 33.77 µL to 202.6 ± 48.91 µL to 203.5 ± 51.6 µL. In contrast, the AVM group initially increased from 192.4 ± 60.83 µL to 201.5 ± 33.94 µL, subsequently declined, and finally rose again to 199.1 ± 51.61 µL (Figure 5C).

In the control group, the E/A ratio peaked at 1.97 ± 0.36 in the 3rd week but dropped dramatically by the 7th week. By the end of the study period, it had returned to a normalized value of 1.75 ± 0.29. The AVM group exhibited varying values throughout the follow-up period (Figure 5D). Both groups exhibited minimal fluctuations in the LA/Ao ratio (Figure 5E).

**Figure 6:**
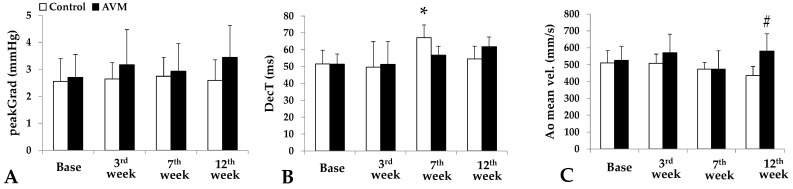
Parameters obtained from the echocardiography recordings preoperatively and on the 3rd, 7th, and 12th postoperative weeks: peak gradient [mmHg]) (**A**), deceleration time (DecT [ms]) (**B**), and aortic mean velocity [mm/s] (**C**). Means ± S.D. * *p* < 0.05 vs. base, # *p* < 0.05 vs. control.

The peak gradient in the control group demonstrated minimal fluctuations. In contrast, the AVM group began at 2.7 ± 0.84 mmHg, increased to 3.17 ± 1.3 mmHg, experienced a slight decline to 2.93 ± 1.02 mmHg, and finally peaked at 3.44 mmHg (Figure 6A).

Regarding deceleration time, the control group peaked at 67.08 ± 7.61 ms in the seventh postoperative week but then normalized to 54.5 ± 7.54 ms by the end of the study period. In contrast, the AVM group showed no significant changes in the third postoperative week, a slight increase in the seventh week, and peaked in the twelfth week at 61.8 ± 5.75 ms from a preoperative value of 51.3 ± 6.11 ms (Figure 6B). Aortic mean velocity significantly increased in the AVM group (Figure 6C).

### 3.2. Hemodynamic Parameters

Hemodynamic parameters (heart rate [bpm], systolic and diastolic blood pressure, as well as mean arterial pressure [mmHg]) did not show significant differences as tested on the 12th week, before extermination and tissue sampling (Table 1).

### 3.3. Morphology of the Shunts

The diameters (O.D.) of the vessels are summarized in Table 2. By the 12th postoperative week, both the arterial and the venous branches of the fistula markedly increased; however, a significant difference was found only in the arterial side (*p* = 0.011 vs. preoperative condition, *p* = 0.025 vs. control side).

### 3.4. Histomorphology

**Figure 7:**
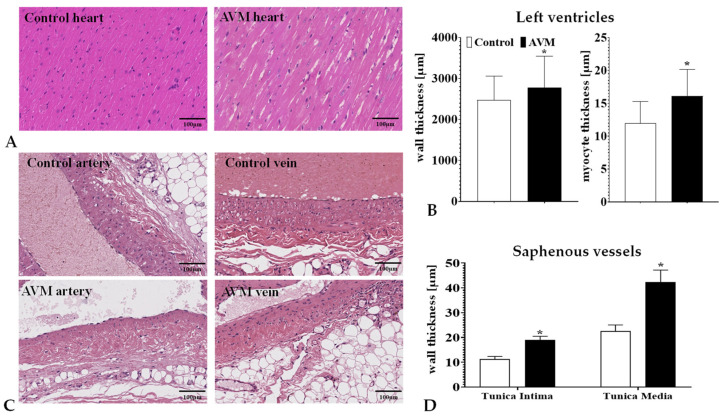
Representative H&E stained histological pictures of the left ventricle (**A**) and the saphenous vessels (**C**) in samples taken on the 12th postoperative week, as well as the thickness of the left ventricle wall layer and cardiomyocytes (**B**) and the vessel wall layers (**D**). H&E staining. Means ± S.D. * *p* < 0.05 vs. control.

The heart wall and myocyte thicknesses across different incision planes (basal, middle, and apical) presented analogous fluctuations after the 12-week observation period, effectively summarized using their combined average. The AVM group exhibited a significant divergence in wall thickness (*p* = 0.0057) compared to the control group. Similarly, an increase in the mean thickness of myocytes was observed in the AVM group (*p* = 0.0001) (Figure 7A).

**Figure 8:**
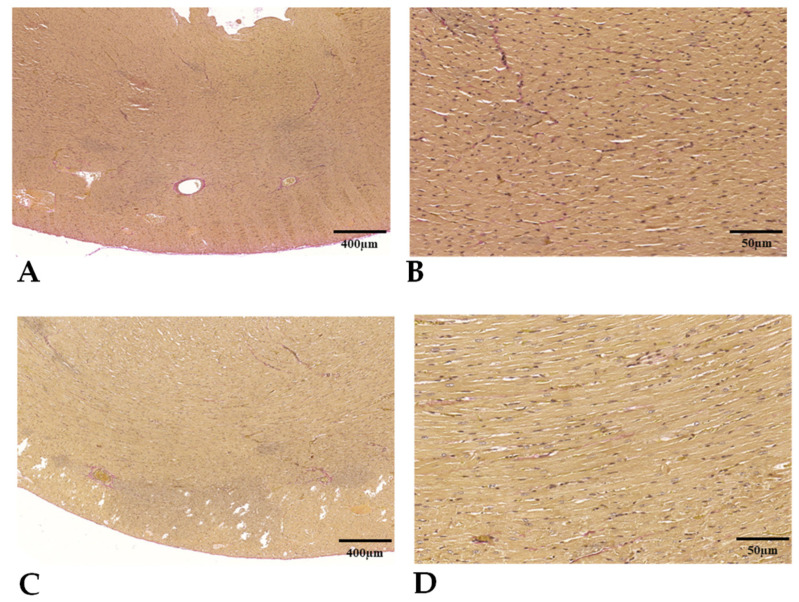
Representative Van Gieson stained histological pictures of the left ventricle in control (**A**,**B**) and AVM group (**C**,**D**).

The qualitative assessment of the H&E and Van Gieson stained sections (Figure 8) did not reveal any signs of myocardial fibrosis stemming from microvascular ischemia or subsequent cellular death in any of the groups. Within the AVM group, cardiomyocytes were noticeably hypertrophic, featuring copious eosinophilic cytoplasm, box-like nuclei, and occasionally exhibiting peculiar forms such as Y-shaped branching. The architectural layout of the myocardium appeared disordered, with cardiomyocyte bundles arranged at both perpendicular and oblique angles to one another, indicative of myocardial disarray.

The tunica intima and media layers of the saphenous vein at the site of the anastomosis significantly enlarged compared to the control saphenous veins (Figure 7B).

## 4. Discussion

The main goal of this work was to establish an AVM model that does not cause prompt and significant hemodynamic and cardiac alterations, compared to other experimental models, using arteriovenous shunts of a larger caliber. Working on rat saphenous vessels needs advanced microsurgical skills but the model may mimic a small, still asymptomatic AVM that can be studied further to investigate the factors that play a role in the progression, enlargement, and destabilization of the AVM.

AVMs are complex vascular anomalies involving a feeding artery and a draining vein and have been known to manifest various symptoms, causing severe neurological complications and, in some cases, disability or death due to hemorrhage [24,26]. Their development is believed to occur during embryogenesis, with potential postnatal occurrences also highlighting the complex nature of their etiology [2,14]. Despite extensive research and significant advancements in medical technology, AVMs’ management still poses considerable challenges due to our limited understanding of their behavior, particularly their reaction to interventions [13,54,55]. 

The carotid-jugular fistula (CJF) model is one of the first models created to study AVMs [37,38]. Spetzler and colleagues pioneered this model to investigate the Normal Perfusion Pressure Breakthrough (NPPB) theory, a phenomenon linked to swelling and hemorrhage in brain tissue surrounding AVM lesions post-surgery [56]. This model was developed in cats and involved an anastomosis between the common carotid artery (CCA) and the external jugular vein (EJV). This anastomosis allowed for non-infarction cerebral hypoperfusion due to retrograde blood flow from the circle of Willis [56]. However, other researchers have found minimal and transient cerebrovascular hemodynamic changes through the CJF formation [57,58], suggesting this model might not fully elucidate NPPB mechanisms.

Morgan and colleagues introduced a modified CJF model in rats involving end-to-end anastomosis of both the CCA and the EJV’s rostral ends. This model showed significant cerebral blood flow (CBF) reduction on the fistula side over 8–12 weeks. Closure of the fistula led to significant CBF elevation, causing blood–brain barrier breakdown under hypertension [44,59]. Rats were primarily chosen for these models due to their economical and accessible nature, despite their anatomical differences compared to humans. Attempts have also been made to create CJF models in monkeys; however, these are challenging to manage, costly, and raise intricate ethical concerns [60].

The carotid-jugular fistula model resulted in prompt hemodynamic alterations across the entire brain, predominantly in the hemisphere on the fistula side; however, it did not significantly impact regional parenchyma. To explore local cerebral hypoperfusion, an intracranial arteriovenous fistula was utilized in a dog model [45]. Dogs were selected for their brain size, which facilitated operations, and their physiological and hemodynamic similarity to humans. After performing a craniotomy, a fistula was established via a femoral venous graft. This graft was anastomosed end-to-side to both the middle cerebral artery’s (MCA) cortical branch and the superior sagittal sinus. Upon opening the shunt, there was a notable reduction in regional cerebral blood flow in the MCA territory, which did not extend to other regions. Upon re-occlusion of the shunt, regional cerebral blood flow swiftly rebounded to pre-opening values within 15 min. There was a significant decrease in regional CO_2_ reactivity upon shunt opening. This animal model’s regional hemodynamic changes effectively mimicked conditions in human brain tissues surrounding AVMs. However, this model was acute, and the procedure was somewhat complex. The extracranial and intracranial arteriovenous fistula models did not include a real AVM nidus. They primarily focused on hemodynamic and pathophysiological changes in the parenchyma adjacent to AVMs rather than on the AVM lesion.

Hence, there has been a necessity for an effective AVM model, one that resembles the human AVM closely in terms of anatomy, physiology, biology, and clinical features to help better comprehend AVM pathogenesis, behavior, and response to treatments.

The major limitation of most of the models using arteriovenous shunts is that, after performing the connections between the arteries and veins, the new situation results in a prompt effect on hemodynamics and cardiac function. So the ‘silent’, asymptomatic AVM cannot be modeled, and the progression and slow enlargement of AVM cannot be followed up.

The cardiac echography results can be explained partly by the aging of the animals, as changes were also seen in the sham-operated control animals. In the presence of slowly ‘maturing’ and enlarging AVMs, moderate but progressive changes were found, and by the 12th postoperative week, relevant histological findings were seen in the heart samples. The thickening of the shunt vessels’ wall was similar to other findings using arteriovenous shunts at different localizations and sizes [47,48]. For instance, in the case of a carotid-jugular fistula, prompt hemodynamic changes were seen and within 6 weeks massive left ventricular hypertrophy appeared [47]. An arteriovenous fistula of different geometries in the femoral region also resulted in significant hemodynamic changes 5 weeks after the operation, while histology of the vessels demonstrated heterogeneously thickened vessel wall layers depending on the distribution of intraluminal pressure and the wall shear stress. Asymmetrical wall shear stress distribution coincided with the intima hyperplasia spots. Changes in the vascular wall can be derived from the altered local blood flow conditions and shearing forces, as confirmed by several previous studies [61].

It was interesting to see that the changes were not uniform or continuously progressive in all the parameters. Adaptation mechanisms in hemodynamics and the cardiovascular system cannot be excluded, as well as the aging and growth of the animals, and possible differences in the motion (however it was not observable) of non-operated and operated legs, all may contribute to the complex changes.

Limitations of this study include the relatively low case number, the effect of regular re-anesthesia, the range of the follow-up period, and the general limitation of extrapolation issues to human clinical practice. 

## 5. Conclusions

The presented rat model of non-congenital, small-caliber AVMs created on saphenous vessels does not cause acute hemodynamic changes versus other models that are based on forming various-sized artificial arteriovenous shunts at different locations. However, in certain cardiac and vascular histomorphology parameters, significant alterations were found by the end of the 12-week follow-up period. The dilatation of the shunted vessels was also developed, confirming the AVM feature. The model seems to be suitable for studying further the progression, enlargement, or destabilization of AVM in different experimental conditions, such as disturbed/altered angiogenesis and vascular remodeling.

## Figures and Tables

**Table 1:** Heart rate, systolic and diastolic blood pressure, and mean arterial pressure 12 weeks after the operation in the control and the AVM groups.

Group	Heart Rate [bpm]	Systolic Blood Pressure [mmHg]	Diastolic Blood Pressure [mmHg]	Mean Arterial Pressure [mmHg]
Control	190.5 ± 16.87	111.74 ± 24.75	94.05 ± 15.2	99.95 ± 18.22
AVM	173.5 ± 41.86	105.65 ± 8.56	82.02 ± 2.77	89.9 ± 2.59

Means ± S.D.

**Table 2:** Outer diameter [mm] values of the saphenous artery and vein before and 12 weeks after the operation.

Localization	Artery [mm]	Vein [mm]
Control preoperatively	0.37 ± 0.02	0.37 ± 0.05
Control side on the 12th p.o. week	0.39 ± 0.05	0.41 ± 0.11
Operated side preoperatively	0.37 ± 0.03	0.38 ± 0.04
Operated side on the 12th p.o. week	0.49 ± 0.06 *^,#^	0.51 ± 0.1

Means ± S.D., * *p* < 0.05 vs. preoperative condition ^#^ *p* < 0.05 vs. control side.

## Data Availability

The data presented in this study are available on request from the corresponding author. The data are not publicly available due to ethical constraints.
